# Supplementary material for: Vascular Morbidity and Mortality in Craniopharyngioma Patients—A Scoping Review
Source: Cancers (Basel). 2024 Mar 8;16(6):1099. doi: 10.3390/cancers16061099 (PMC10969212; doi:10.3390/cancers16061099)
Supplement: Supplementary file 1 [file cancers-16-01099-s001.zip › cancers-2873456-supplementary.pdf]

Table S1: Data extraction table.

Reference	Title	Study type	Study size (n)	Childhood	Adult	Study period	Vascular complications	Etiology	Time since CP treatment (follow-up)	Outcome	Result
Aboukais et al. 2021	Fusiform dilatation of internal carotid artery after pterional but not subfrontal craniotomy in 6 patients	case report	4	x		na	FDICA	Surgery	5-12 months	n.a.	delay between surgery and FDICA diagnosis: 5-12 months in CP; one case ICA bifurcation involvement; two patients with FDICA progression (16-24 mm diameter)
Almeida et al. 2019	Moyamoya Syndrome after Radiation Therapy: a Clinical Report	case report	1	x		na	Moyamoya syndrome	Radiation	20 years	n.a.	36 yo woman, presented with disorientation and imperceptible speech lasting for 1 hour, no stroke or hemorrhage, history of CP, radiotherapy
Bitzer et al. 1995	Progressive cerebral occlusive disease after radiation therapy	case report	1	x		na	Moyamoya syndrome	Radiation	6 years	n.a.	19-year-old woman presented with recurrent ischemic brain lesions after radiation therapy for treatment of a craniopharyngioma during childhood. Cerebral angiography 6 and 12 years after completion of radiation therapy revealed progressive cerebral arterial occlusive disease involving the internal carotid artery on either side of the circle of Willis, with

											abnormal netlike vessels and transdural anastomoses (moyamoya syndrome)
Boekhoff et al. 2021	Cerebral Infarction in Childhood-Onset Craniopharyngioma Patients: Results of KRANIOPHARYNGEOM 2007	retrospective cohort study	244	x		2007-2019	Stroke	Surgery	0.5-53 days	incidence	28 of 244 (11%) had cerebral infarction after surgery for childhood-onset CP
Bolsi et al. 2020	Pencil beam scanning proton therapy for the treatment of craniopharyngioma complicated with radiation-induced cerebral vasculopathies: a dosimetric and linear energy transfer (LET) evaluation	case report	16	x		2008-2014	Moyamoya syndrome	Radiation	19 (14-24) mo	incidence	2 out of 16
Boon et al. 2016	When Occam's razor fails: hemipontine infarct on a background of previous surgery and radiotherapy for craniopharyngiom	case report	1		x	na	hemipontine infarct	Radiation	13 years	incidence	68 years old patient, with CP surgery and RT 13 years earlier; treatment: testosterone, growth hormone, hydrocortisone, levothyroxine, desmopressin and metformin for type 2 diabetes mellitus

	a										
Bülow et al. 1998	Postoperative Prognosis in Craniopharyngioma with Respect to Cardiovascular Mortality, Survival, and Tumor Recurrence	retrospective cohort study	60	x	x	1951 - 1988	myocardial infarction, heart failure, pulmonary embolism, atherosclerosis univ.,	not specified	12.5 yr (1 d to 40 yr)	cardiovascular (and cerebrovascular) mortality	SMR (3.21; 95% CI, 1.29–6.61); without early-postoperative: SMR, 3.22; 95% CI, 1.29–6.63)
Cavallo et al. 2013	The endoscopic endonasal approach for the management of craniopharyngiomas involving the third ventricle	case report	12	x	x	2001-2011	postsurgical complications: hemorrhage, CSDH	Surgery	5-63 months	incidence	2/12 cases had chronic subdural hematoma
Chandrakasan et al. 2011	Risk factor and Managment of Deep Venous Thrombosis in Children	case report	3	x		na	deep venous thrombosis	not specified	13 years; 2 weeks; 2 months	n.a.	multiple pathophysiological pathways following CP surgery in these female CP patients: oral contraception, family history (16 years old), post-surgical complication (13 years old); hypernatremic dehydration (5 years old)

Cho et al. 2012	Vasculopathy after intracystic bleomycin administration for a recurrent cystic CP	case report	1	x		na	cerebral infarction	Surge ry	4 months	n.a.	28 months years old boy, GTR, intracystic bleomycin administraion; 3 mg for 14 weeks; four months later hemiparesis; enlarged tumor cyst and infarction at the territory of left MCA
Edmonsto n et al. 2022	Limited surgery and conformal photon radiation therapy for pediatric craniopharyngiom a: long-term results from the RT1 protocol Drucilla	cohort study	101	x		199 8-201 3	vasculopath y	Radia tion	1.05 years (range 0.56–4.86 years)	incidence	2 patients with perioperative strokes involving middle cerebral circulation, developed permanent deficits; abnormal MRA (n=15); venous sinus thrombosis (n = 1), transient ischemic attack (n = 1), heart valve replacement (n = 1), dyslipidemia (n = 1), and abnormal cerebral angiography (n = 2), empiric after stroke-like symptoms without imaging correlate (n = 2); cumulative incidence of vasculopathy was 7.93 ± 2.71% at 10 years; PTV and sex are associated with the incidence of vasculopathy
Elliott and Wisoff 2010	Fusiform dilation of the carotid artery following radical resection of pediatric CP	retrospective cohort study	86	x		198 6-200 6	FDCA	Surge ry	10.5 (SD 8.3) years for FDCA patients	Incidence	7 of 86 patients had FDCA

Ferraù et al. 2017	Visceral adiposity index as an indicator of cardiometabolic risk<br>in patients treated for craniopharyngioma	retrospective cohort study	24	x	x	1990-2015	cardiometabolic and cardiovascular risk assessment	Obesity	15.9 (SD 9.3)	ASCVD risk scores (10 years and lifetime)	overall (10 years; lt): 3.7 (SD 6.2); 38.2 (SD 12.5); CO: 0.3 (0.4), 35.5 (12.9); AO: 9.5 (7.4), 47.2 (5.5)
Fischer et al. 2004	Inflammatory thromboembolic complication after C surgery	case report	1		x	na	cerebral infarction in left MCA territory and meningitis	Surgery	10 days	n.a.	30 years old patient with visual impairment and DI, CP surgery (GTR), ACP, meingitis on day 5 post surgery, 10 days after surgery stroke
Fukuhura et al. 2017	Acute subdural hemetaoma immediately after extended transsphenoidal surgery for CP	case report	1		x	na	subdural hemorrhage	Surgery	30 minutes	n.a.	79 years old female patient, presented with depression and appetite loss, diagnosed with CP, panhypopit, DI, underwent TSS and had immediate acute right subdural hematoma; bleeding from the vein of Labbé
Gleeson et al. 2008	Do no harm': management of CP	case report	1	x		na	Moyamoya syndrome syndrome	Radiation	1 year	n.a.	11 years old boy presented with visual impairment, vomiting, headaches; preoperative fatigue but no growth retardation; appetite unaltered but more drinking, hypothyroidism, hydrocortisone replacement; aimed GTR, 6-week course of external beam RT because of residual tumor; post-surgery: DI, massive weight gain, behavioural problems; hospital

											admissionbecause of diabeteic ketoacidosis, then cerebral angiogram revelaed widespread stenosis of the arteries aroud the circle of Willis and moderate Moyamoya appearance
Hall et al. 2018	Risk of Radiation Vasculopathy and Stroke in Pediatric Patients Treated With Proton Therapy for Brain and Skull Base Tumors	retrospective cohort study	135	x		2006-2015	Vasculopathy and stroke	Radiation	3 years (0.1-9.6)	incidence	26 of 135 patients with CP developed vasculopathy (more often than other entities, but not significantly different)
Hawkins et al. 1990	Late deaths after treatment for childhood cancer	retrospective cohort study	49	x		before 1971	Tumor-related death	Tumor	min. 5 years	mortality	10 patients with CP in tumor-related death group (n=49); one patient had cerebroartherosclerosis after RT; another patient intracranial venous thrombosis after surgery for recurrence
Hoffmann et al. 2016	Fusiform dilatation of the internal carotid artery in childhoodonset craniopharyngioma: multicenter study on incidence and long-term outcome	retrospective cohort study	583	x		2001-2015	FDCA	Surgery	8.0 (0.1-38.9) years; appearance of FDCA between 0.1-5.6 years	incidence	14 (2.4%) CP patients had FDCA after surgery; Vascular complications such as hemorrhage or thrombosis were not observed in any FDCA during long-term follow (median: 9.7 years, range 3.3–23.8 years).

Holmer et al. 2009	Hypothalamic involvement predicts cardiovascular risk in adults with childhood onset craniopharyngioma on long-term GH therapy	cross-sectional study	42	x		1958-2000	CVD (and risk factors)	Tumor	20 (4-40) years	prevalence	The criteria for'medium-high CVD risk', predictors of diabetes mellitus and cardiovascular events, were met by 55-60% of female craniopharyngioma patients compared to 10-20% of controls.<br>There was no difference in 'CVD risk markers' between male craniopharyngioma patients and controls.<br>The greater 'CVD risk' and insulin levels among females in the TGTV group compared to females in the non-TGTV group indicated that hypothalamic injury from the tumor was more important than female gender risk.
Indelicato et al. 2017	Clinical outcomes following proton therapy for children with central nervous system tumors referred overseas	cohort study	45	x		2008-2016	stroke	Radiation	median: 2.6 years (range, 0.2–7.6 years)	incidence	2 out of 45 patients with CP
Jamaluddin et al. 2020	Visual Deterioration due to unripted saccular internal carotid artery aneurysm in an operatied case of	case report	1		x	-	supra-clinoid carotid aneurysm	Surgery	4 years initital; then 16 years	n.a.	after 4 years: supra-clinoid carotid aneurysm; after 16 years after surgery wide-necked saccular dorsal wall aneurysm

	CP										
Jung et al. 2020	Autonomic Dysfunction is Associated with Increased Cardiometabolic Risk in Patients with childhood-onset CP	cross-sectional study	53	x	x	2014-2016	cardiac autonomic function; heart rate	Obesity	mean 10.8 (SD 5.5)	standard devioation of all normal R-R intervals; total power, rott mean square of the difference of successive R-R intervals, high frequency, low frequency, LF/HD ration	Patients with the MetS showed lower levels of overall variability (SDNN and TP), parasympathetic modulation (RMSSD and HF) than those without the MetS. As overall HRV decreased, HOMA-IR, triglycerides and systolic BP significantly increased, after adjusting for covariates. decreases in HRV and parasympathetic modulation were independent risk factors for the MetS in patients with childhood-onset craniopharyngioma.

Jung et al. 2021	Cardiac autonomic dysfunction is associated with hypothalamic damage in patients with childhood-onset craniopharyngioma	cross-sectional study	48	x	x	2014-2016	cardiac autonomic function; heart rate	Tumor	mean 10.5 (SD 4.8)	standard devioation of all normal R-R intervals; total power, rott mean square of the difference of successive R-R intervals, high frequency, low frequency, LF/HD ration	Patients with extensive HI demonstrated lower means of overall variability (SDNN and TP), parasympathetic modulation (HF), and sympatho/sympathovagal modulation (LF) markers than those with mild HI. Centrally obese patients showed a lower mean parasympathetic modulation index (HF) than the centrally non-obese. The interaction of HI and central obesity on the HRV indices was not significant.
Kato et al. 2019	Efficacy of Direct Revascularization Surgery for Hemorrhagic Moyamoya Syndrome As a Late Complication of Cranial Irradiation for Childhood Craniopharyngioma	case report	1	x		na	Moyamoya syndrome syndrome	Radiation	30 years	n.a.	36 yo man from Japan, intraventricular hemorrhage from a pseudoaneurysm at the extended left anterior choroidal artery as an abnormal collateral of MMS; admission due to sudden headache; microsurgical removal of the craniopharyngioma by bifrontal craniotomy and subsequent fractionated cranial irradiation with a total radiation dose of 50 Gy.

											There had been no tumor recurrence for 30 years; therapy with STA-MCA bypass
Koumas et al. 2019	Outcomes following endoscopic endonasal resection of sellar and supresellar lesions in pediatric patients	case report	12	x		2010-2015	Vasospasm, Stroke	Surgery	mean fup 39 months	incidence	3 cases (25%) with Vasospasm/stroke during examination period; Three patients (10%) had vasospasm/stroke, all after craniopharyngioma resection. This involved one patient with silent stroke of right thalamic region, one patient with thalamic stroke causing decreased level of consciousness requiring 3-month inpatient stay, and one patient with vasospasms leading to large watershed stroke proved by magnetic resonance angiography.

Kralik et al. 2017	Radiation-Induced Large Vessel Cerebral Vasculopathy in Pediatric Patients With Brain Tumors Treated With Proton Radiation Therapy	case report	14	x		2007-2014	radiation-induced large vesselcerebral vasculopathy (RLVCV)	Radiation	3.8 years and 1 year	incidence	among 4 patients with RLVCV, two had a CP. Patient 1: 54gy, sella and suprasellar cistern, acute right MCA and right ACA infarct; treated with bilateral pial synangiomatosis. Patient 2: 54 Gy, 1 y since diagnosis, suprasellar cistern, acute right MCA and ACA infarcts; right ICA, MCA and ACA stenosis (severe), no collaterals, no treatment
Lakhanpal 1995	MR and CT diagnosis of carotid pseudoaneurysm in children following surgical resection of craniopharyngioma	case report	2	x		na	carotid pseudoaneurysm	Surgery	na and 5 months	n.a.	Case 1: 14 y old boy, GTR, progressive fusiform aneurymal dilatation of the right supraclinoid carotid artery; Case 2: 12 y old, subtotal resection, 5500 cGy RT, five months post surgery, recurrent tumor by CT and another surgery was made; fup CT, MRI, MRA and angiography 16 m after inital surgery confirmd presence of pseudoaneurysm of the right supraclinoid carotid artery. 28 m after inital surgery, no furhter enlargement was found

Lambert et al. 2022	Venous thromboembolism in the setting of pediatric central diabetes insipidus: a systematic review of the literature and report of 2 cases	case report	2	x		na	pulmonary embolism (following deep venous thrombosis)	Surgery	25 days after surgery and 17 days after surgery	n.a.	Case 1: 10 yo boy, near-total resection for CP, postperative central DI, admission for pseudomeningocele, cradiac event, VA-ECMO required, diagnosis: pulmonary embolism after DVT; Case 2: 4 yo boy, GTR for CP, postoperative central DI, on POD 17 dyspenic, bilateral pulmonary embolism, no DVT
Lee et al. 2011	Moyamoya Syndrome Precipitated by Cranial Irradiation for Craniopharyngioma in Children	case report	2	x		na	TIA and Moyamoya syndrome	Radiation	2 years and 3 years	n.a.	Case 1: 13 yo girl, subtotal resection, whole-brain RT; 2 years later surgery for recurrence, at surgery occlusion of both distal ICAs, transdural meningocortical anastomosis; Case 2: 4 yo girl, subtotal CP removal, post-operative RT, 3 years after RT lower extremity weakness, MRI showed bilateral supraclinoidal ICA occlusion, with severe narrowing of the right ACA and MCA, died 7 mo later due to adrenal insufficiency
Lee et al. 2017	Proton therapy in paediatric oncology: an Irish perspective	retrospective cohort study	16	x		before 2015	Moyamoya syndrome	Radiation	na	incidence	1 out of 16

Li et al. 2015	Endovascular Treatment for Fusiform Dilation of Internal Carotid Artery Following Craniopharyngioma Resection: A Case Illustration	case report	1	x		na	Fusiform dilation	Surgery	2	n.a.	13 yo boy, headache for two years; CP; GTR; GH for 2 months; MRI after 1y tumore recurrence; gamma-knife surgery 50 Gy; slow growth; on MRI the aneurysm; CT confirmed Fusiform dilation of internal carotid artery; stent and coils for treatment; boy recovered well; no headaches; endocrine replacement; growth
Linfante et al. 2008	Fusiform Dilatation of the Internal Carotid Artery After Craniopharyngioma Resection	case report	1	x		na	Fusiform dilation	Surgery	5		20 yo old woman; presented with severe headache, nausea, and vomiting to ER; magnetic resonance angiography revealed a fusiform dilatation of the right internal carotid artery; Cerebral angiography confirmed the lesion; headache was diagnosed as a migraine and she was successfully treated with medication; no symptoms at 6 months and 1 y fup
Liu et al. 2009	Vascular Abnormalities in Pediatric Craniopharyngioma Patients Treated With Radiation Therapy	retrospective cohort study	22	x		1995-2008	vasculopathy	Radiation	44 months (range 4–85 months)	incidence	Six of the 20 were found to have some type of vasculopathy. One had bilateral temporal cavernomas, one had moyamoya syndrome, one had an aneurysm of the internal carotid artery and three children had decreases in the caliber of

											the carotid or cerebral arteries, but were asymptomatic. Two of the six children with abnormalities also received intracystic bleomycin prior to radiation therapy.
Liu et al. 1991	Fusiform Aneurysm After Surgery for Craniopharnygioma	case report	1		x	na	Fusiform dilation	Surgery	4 months; 3 years	n.a.	24 yo woman; craniotomy; GTR; after 4 mo Dilatation of the right supraclinoid segment of the ICA; after 3 y aneurysm elarged; clipping; the ICA was diffusely enlarged from the distal dural ring of the cavernous sinus to the carotid bifurcation with no evidence of focal vessel wall injury
Lo et al. 2016	Cross-Sectional Cohort Study of Cerebrovascular Disease and Late Effects After Radiation Therapy for Craniopharyngioma	cross-sectional study	20	x		1971-2010	stroke	Radiation	13.7	incidence	six of 19 (32%) patients had stroke (ischeamic, heamorrhagic); 1 already before RT (post-surgery)

Lucas et al. 2022	Pre- and Post-therapy Risk Factors for Vasculopathy in Pediatric Craniopharyngioma Patients Treated with Surgery and Proton Radiotherapy	cohort study	94	x		2011-2016	vasculopathy	Radiation	median of 7 years (range, 4.92–9.36).	incidence	post surgery (pre RT): 9 stenosis, 4 infarction, 3 aneurysms; 34 (36.3%); new post-RT vascular abnormalities: 22 stenosis, 2 aneurysms, 3 cavernoma, 3 dilated perivascular space, 3 infarctions
Macdonald 1997	Subarachnoid hemorrhage and vasospasm following removal of craniopharyngioma	case report	1	x		na	hemorrhage	Surgery	8 hours after surgery for recurrence	n.a.	7 yo patients, STR, RT post surgery, regrowth of tumor 2 yars later (1992), partially resection, subarachnoid hemorrhage on CT 8 h after surgery, surgery, clot removed, further complications, severely diasabled three months post OP
Merchant et al. 2023	Proton therapy and limited surgery for paediatric and adolescent patients with craniopharyngioma (RT2CR): a single-arm, phase 2 study	cohort study	94	x		2011-2016	vasculopathy	Radiation	median 7.62 years (IQR 6.48–8.54)	incidence	7 in PTB and 7 in XRT with vasculopathy during fup; 3-year cumulative incidence 3.98% (SE 1.96) [XRT] and 4.49% (SE 2.21) [PTB]; 5-year cumulative incidence 4.99% (SE 2.19) [XRT] and 7.87% (SE 2.87) [PTB]

Mitchell et al. 1991	Stroke as a late sequela of cranial irradiation for childhood brain tumors	retrospective cohort study and case report	1	x		199 1	stroke	Radia tion		incidence	One death; cerebral infarction; craniopharyngioma diagnosed at age 4 years, in 1972. only partially resectable; treated with a total of 6650 rads to a port including the sella, suprasellar region, hypothalamus, anterior temporal lobes, and inferior frontal lobes. Two years after treatment, mild right hemiparesis, which worsened over the next 3 weeks; right hemiplegia and aphasia; she was awake but aphasic and hemiplegic; difficulty chewing and swallowing. The following morning, cerebral angiography was performed, revealing occlusion of the right internal carotid artery, and marked narrowing of the left internal carotid artery, middle and anterior cerebral arteries, and right posterior communicating artery. Two days later, she experienced the onset of left-sided focal motor seizures. The following day, she exhibited decerebrate posturing, central hyperventilation, and coma, followed by hypotension, bradycardia, and death. Autopsy revealed bilateral cerebral infarctions, as well as diffuse gliosis. Branches of the carotid

											artery showed very severe thickening, with subintimal proliferation of connective tissue (Figure 3). Smaller vessels showed cellular intimal proliferation, with areas of leukocytic infiltration.
Nagata et al. 2010	Pathological findings of fusiform dilation of the internal carotid artery following radical dissection of a craniopharyngioma	case report	1	x		na	Fusiform dilation	Surgery	2 months	n.a.	5 yo boy, sudden consciouness disturbance, huge cystic CP, before Ommaya reservoir and ventricle drainage, aggressive resection of the tumor, cyberknife radiation for residual 6 mo after surgery; tumor continued to grow; FDICA was compressing optic nerve
Nash et al. 2016	Management strategy for treatment of vasospasm following transspenoidal exiciion of craniopharyngioma	case report	2		x	na	Cerebral vasospasm	Surgery	11 days and 5 days	n.a.	Case 1: 48 yo female, papillary CP, transspenoidal resection, no sign. Intra-operative bleeding or vascular injury, represented on day 11 post OP with dysphasia, lethargy, unsteadiness and hemiparesis; serum sodioum was 148 mm/l, blood lab normal; on MRI restricted diffusion in the basal lobes, the anterior limb of the left internal capsule, and the left lobe; MRA

											naroowing of supraclinoid carotid arteries and the proximal A1/M1 segments bilaterally; treatment for clinical vasospasm; good clinical neurological recovery, treated for DI; Case 2: 49 yo female, adam. CP, transsphenoidal surgery, minmal intra-operative bleeing, no significant vascular injury, no improvement in visual field after surgery, re-operation at day 1 for the fat graft, DI, SIADH; on day 5 post surgery dyphasia and reduced conciousness level; sodium slightly low with 132 mmol/l; CT angiogramm showed proximal vasospasm affecting the iternal carotid and middle cerebral arteries; systematic hypertension and hypervolaemia treatment; after 24h no improvement, CT scan revealed infarction; cerebral angiopathy, confirmed vasospasm, underwent bilateral balloon angioplasty; slow recovery and rehabiliation
Nishioka et al. 2000	Repeated Hemorrhage in Ciliated Craniopharyngioma	case report	1		x	na	intratumoral hemorrhage	Tumor	22 years	n.a.	49 yo female, severe headache, nausea, left visual disturbance, subtotal resection transcranially, lost right visual function, no signs of recurrence, normal pit function, no examination for 10

											y; on admission: general fatigue, excessive sleep requirement, nausea, anorexia, cold intolerence, dizziness, suggestive of hypopit, neurological: left blurred vision, temporal hemianopsia, lab confirmed hypopit, recurrence of pap. CP revleaed through MRI; resurgery; tumor included many thin dilated blood vessels
Olsson et al. 2015	Excess Mortality and Morbidity in Patients with Craniopharyngioma, Especially in Patients with Childhood Onset: A Population-Based Study in Sweden	population-based study	307	x	x	1997-2011	circulatory diseases, ischemic heart disease, cerebrovascular disease	not specified	mean 9 years (0-25)	SMR, SIR	SMR circulatory diseases 3.6 (95% CI 2.1-5.7); SMR ischemic heart disease 3.6 (95 % CI 1.6-6.8); SMR cerebrovascular 5.1 (95% CI 1.5-12); SIR cerebral infarction for CCP 365 (95% CI 194-624)
Pereira Cruz 2002	Intracranial Aneurysm and Vasculopathy after Surgery and Radiation Therapy for Craniopahryngioma: Case Report	case report	1	x		na	ICA aneurysm and vasculopathy	Radiation	5 years	n.a.	at diagnosis 14 years; female; giant cystic CP; pterional appraoch; complete recovery; panhypopit; external RT with 54 Gy; clinically unremarkable; 5 years later in regualr MRI: right ICA bifurcation aneurysm, confirmed by angiography, watchful waiting, aneurysm was reduzed in size

Pereira et al. 2005	High prevalence of long-term cardiovascular, neurological and psychosocial morbidity after treatment for craniopharyngioma	cohort study	54	x	x	1965-2002	cerebrovascular accident, myocardial infarction, TIA	not specified	10 years	prevalence, mortality	22% total CV morbidity, 14% CVA, 2% TIA, 6% MI; CVA more prevalent in premenopausal oestrogen-deficient women; mortality: 40% were CV complications
Pritz et al. 2002	Reuptured True Posterior Communicating Artery Aneurysm and Cystic Craniopharyngioma	case report	1		x	na	Aneurysm	Tumor	at surgery	n.a.	49 yo female, headaches, memory loss, altered mentation; ventricle cystic mass, shunt, right sided pteroinal craniotomy, cyst reoccurred, RT, another craniotomy, aneurysm was treated through surgery, stroke, incomplete recovery
Probst et al. 1992	Herzstillstand bei Exstirpation großer Kraniopharyngeome: 2 fälle mit erfolgreicher Reanimation - Überlegung zur Pathogenese	case report	2	x	x	na	cardiac arrest	Surgery	during surgery	n.a.	two cases: 8 yo female, large-sized CP, post-surgical caridac arrest, reanimation for 40 minutes, full recovery; 21 yo female, large-sized CP, during surgery, reanimation for 5 minutes, recovery, radiotherapy

Qian et al. 2019	Intraoperative Finding of an Anterior Communication Artery Blister-Like Aneurysm During a Primary Craniopharyngioma Resection: Accidental or Incidental?	case report	1		x	na	Aneurysm	Tumor	during surgery	n.a.	41 yo male, two months history, right frontal craniotomy, blister-like anuerysm of the anterior communicating artery, GTR, suturing technique for aneurysm afterwards; local vascular cytotoxic and inflammatory reaction due to lack in cyst and cyst fluid
Qiao et al. 2023	Machine learning prediction of venous thromboembolism after surgeries of major sellar region tumors	cohort study	371		x	2018-2021	venous thromboembolism (PE and DVT)	Surgery	1 to 58 days after surgery	incidence	30 (24.2%) of 371 cases with CP had VTE; Patients with increased age, specific tumor pathology (craniopharyngioma and chordoma), craniotomy, CSF leakage and long surgical duration were at a higher risk of developing VTE.
Ravindra 2021	Comparison of multimodal surgical and radiation treatment methods for pediatric craniopharyngioma: long-term analysis of progression-free survival and morbidity	cohort study	63	x		1997-2018	Moyamoya syndrome, stroke	Radiation	STR + PBT: 3.95 (1.91–7.03) and GTR: 6.5 (5.03–8.6) years	incidence	2 cases with moyamoya (STR+PBT), 1 patient with postoperative stroke (GTR) subsequent strokes

Reynolds et al. 2016	Radiation-Induced Moyamoya Syndrome after Proton Beam Therapy in the Pediatric Patient: A Case Series	case report	1	x		na	Moyamoya syndrome	Radia tion	2 years	n.a.	5 yo female, 3-week history of headaches, craniotomy, incomplete, transient, leftsided paresthesia and weakness, hypothalamic syndrome
Ricarte et al. 2015	Symptimatic Cerebral Vasospasm and Delayed Cerbral Ischemia Following Transsphenoidal Resection of Craniopharyngio ma	case report	1		x	na	Vasospasm, Stroke	Surge ry	2 weeks	n.a.	67 yo female, transsphenoidal resection, 2 weeks later presented with drowsiness, repeated CT 48 h after the initial symptoms bilateral infarcts in the terriotory of ACA, vasospasm, CT angiography confirmed, patient remained in ICU, discharge after 2 weeks, one month later still mild paraparesis, fup head CT showed bilateral ACA infarcts

Sandvik et al. 2019	Vascular complications in pediatric craniopharyngioma patients: a case-based update	case report	2	x		na	Fusiform aneurysm, Moyamoya syndrome syndrome	Radiation	12 years and 3 years	n.a.	Case 1: AaD 5 y, female, cystic CP, hydrocephalus, transnasal fenestration of the cyst, residual tumor, RT, after 7 years progression, proton beam therapy (54 Gy), cystic progression in th efollowing year, 2 years later another cystic progression, ommaya resevoir, interferon- alapha treatment, sudden visual impairment, slightly improvement of vision, after 12 years sudden headache, MRA found fusiform aneurysm at the suprior part of the right carotid siphon, vasculitis treatment, over 8 motnhs slight growth of aneurysm, endovascular flow diverter, persistent contrast circulation, most recent follow up (6 motnhs after treatmen), slight reduction of aneurysm; Case 2: AaD 6 years, female, endoscopic cyst puncture, three wks repetiion of procedure, solid tumor removed, posoperative leakage of cerebrospinal fluid, two revision surgeries, possible vasospasm, DSA showed normal appearance of the right carotid siphon and middle cerebral artery, fup MRI showed progress in cyst size, sterotactic gamma knife lesion one year

											after diagnosis, 3 years after diagnosis intermittent dysarthria, left-sided hemidystonia, MRA showed right-sided carotid stenosis, moyamoya syndrome, reduced blood flow in right hemisphere, DSA revealed occlusion of the right carotid artery and vascular supply from chorodial collateral vessels, right sided temporal artery pial synangiosis, post-OP contralateral hemiparesis, hemorrhagic infarction, 18 months after surgery, recovery from hemidystonia, but impaired sensibility in left side
Sarkar et al. 2022	Upfront adjuvant irradiation versus postoperative surveillance following incomplete surgical resection of craniopharyngiomas in children and young adults	retrospective cohort study	37	x		2008-2018	vasculopathy	Radiation	64.7 months (range, 14–134 months)	incidence	Two children in the adjuvant RT group developed asymptomatic radiation-related vasculopathies on follow-up; both had had received multiple interventions, including both surgery and salvage RT.
Singh et al. 2017	Vasospasm after craniopharyngioma surgery: can we prevent it?	case report	60	x	x	4 years	Vasospasm	Surgery	2-3 weeks	incidence	6 out of 60 patients had vasospasm; only 2 of them favourable outcome

Sowithayasakul et al. 2021	Cardiac remodeling in patients with childhood-onset craniopharyngioma - results of HIT-Endo and KRANIOPHARYNGEOM 2000/2007	cohort study	36	x		2015-2016	cardiac status: TTE parameters	Obesity	median 8.8 years	TTE parameters	degree of obesity in CP is correlated with an increased left ventricular wall thickness (IVSd and LVPWd) (r=0.645, p<0.001)
Strenger et al. 2008	Intracerebral Cavernous hemangioma after Cranial irradiation in Childhood	retrospective cohort study	171 (mixed tumors)	x		1980-2003	intracerebral cavernoma	Radiation	10 years	incidence	1 patient with CP developed intracerbral cavernoma: female, 55 Gy RT
Sutton et al. 1994	Vascular Complications of Surgery for Craniopharyngioma and Hypothalamic Glioma	case report	9	x		1982-1993	Fusiform dilatation of carotid artery (FDCA)	Surgery	5-11 years	n.a.	follow-up of patients with FDCA; n.a. required treatment or have experienced hemorrhage; one patient died due to recurrent tumor, all other alive
Sutton et al. 1991	Fusiform dilatations of the carotid artery following radical surgery of childhood craniopharyngiomas	case report	9	x		1982-1990	Fusiform dilatation of carotid artery (FDCA)	Surgery	6-18 months	incidence	9 (29%) of 31 children wih CP had FDCA after surgery

Takeuchi et al. 2012	Anterior cerebral artery dissecting aneurysm associated with untreated craniopharyngioma	case report	1		x	na	Fusiform diltatation of anterior cerebral artery (ACA)	Tumor	at admission	n.a.	49 yo male, with untreated CP, admitted due to repeated transient left hemiparesis, CP incidental finding, FDCA on CT, cranioptomy, treatment for aneurysm, full recovery
Tirakotai et al. 2002	Successful management of a symptomatic fusiform dilatation of the internal carotid artery following surgery of childhood craniopharyngioma	case report	1	x		na	Fusiform diltatation of the internal carotid artery (FDICA)	Surgery	2 years after last surgery (progress); 4 years after initial	n.a.	10 yo female, STR, progress, two years later progressive headache, no progress, giant fusiform aneurysm of the right carotid artery, microsurgical clipping, postoperatively full recovery
Ujifuku et al. 2010	Malignant Transformation of Craniopharyngioma Associated with Moyamoya Syndrome	case report	1		x	na	Moyamoya syndrome syndrome	Tumor	5 years	n.a.	32 yo male, incomplete resection, gamma knife radiosurgery, loss of vision, progress 5 y after inital surgery, carotid angiography demonstrated bilateral ICA occlusions, moyamoya vessels, ztansphenoidal biopsy, hard and bony tumor, prone to bleed, odontogenic component, keratinization, conventional radiotherapy, biopsy, malignant components in tumor, chemotherapy planned, ischaemic attack, died 43 days after last surgery

Verweij et al. 2021	Cardiovascular risk profile in Growth Hormone treated adults with a cranioharyngioma compared to a non-functioning pituitary adenoma: a national cohort study	retrospective cohort study	291		x	1998-2009	cerebrocascular accidents (CVA): stroke and TIA, CVD: hypertension, angina pectors, myocardial infarction, intermittent claudication, heart failure, atrial fibrillation, hypertrophic cardiomyopathy, cardiac and periphal vascular surgery	Obesity	? "long fup"	incidence HR (95% CI) compared to NFPA patients	CVA: 0.99 (0.19-5.10); CVD: 1.17 (0.57-2.39); Death: 1.1 (0.21-5.69)
Visser et al. 2010	Late mortality in pediatric patients with craniopharyngioma	retrospective cohort study	41	x		1967-2003	MCA infarct after Moyamoya syndrome disease	Radiation	median fup 8.71 (0.9-25.6) years	mortality	massive middle cerebral artery infarct secondary to radiation induced Moyamoya disease in 1 out of 41 cases

Wang et al. 2016	Bypass surgery to treat symptomatic fusiform dilation of the internal carotid artery following craniopharyngioma resection: report of 2 cases	case report	2	x		na	Fusiform dilation of the internal carotid artery (FDICA)	Surgery	5 years	n.a.	Case 1: 15 yo male, 2-y history of right-sided visual dificits, intact neurological fcuntion, GTR for CP, right pterional appraoch, 5.5. y erlier, recurrence 3 y after fup, without vascular abnormality, MRI dilation of the right supaclinoid segment of the iCA, supasellar lesion consistent with recurrent CP, surgery, total removal of CP, disappearence if the FDICA, replacement medication, intact neruological function; Case 2: 13 yo female, 3 motnh history of severe headache, >5 y ago GTR for CP, right frontotemporal cranioptomy, uneventful post-op, no neurological deficits, free thyorxin midly deficient, dilated right ICA, no tumor recurrence, FDICA extending from the posterior communicating artery to the bifurcation of the ICA, bypass surgery, uneventful post-op, temporal hematoma, replacement therapy, intact neurological function

Wijnen et al. 2018	Excess morbidity and mortality in patients with craniopharyngioma: a hospital-based retrospective cohort study	retrospective cohort study	224	x	x	1987-2014	Circulatory diseases	not specified	median fup 13 years (IQR 6-12)	mortality	circulatory diseases (ICD-10 chapter 9) (SMR: 2.3, 95% CI: 1.1–4.5) - mainly due to cerebrovascular disease
Wu et al. 2016	Incidence and long-term outcome of postradiotherapy moyamoya syndrome in pediatric patients with primary brain tumors: a single institute experience in Taiwan	retrospective cohort study	391 (mixed tumor)	x		1975-2005	Moyamoya syndrome syndrome	Radiation	median 7.3 years	incidence	3 out of 24 patients with CP identified with moyamoya; incidence rate: 0.013/person-year; no person died
Yamashita et al. 2004	Craniopharyngiomas With Intratumoral Hemorrhage	case report	2		x	na	intratumoral hemorrhage	Tumor	at diagnosis	n.a.	Case 1: 22 yo male, meningitis, declining vision, bitemporal hemianopsia, DI, tumor from the intra- to the suprasellar region with hemorrhage, craniotomy, total extirpation of tumor wihtin the hemorrhage, PCP, hormonal replacement, no recurrence; Case 2: 29 yo female, bitemporal hemianopsia, tumor extending from intra- to suprasellar region, transsphenoidal surgery, incomplete, normalized vision,

											readmission with severe headache, hemianopsia on the right (2 motnhs post OP), emergent craniotomy, rapid visual impairment, hemorrhagic tumor, partially removed, hemetoma, PCP diagnosis, no keratinization, visueal field improved post-op, gamma-knife therapy for residual tumor, no re-growth
Yu et al. 2016	Retrospective analysis of 14 cases of remote epidural hematoma as a postoperative complication after intracranial tumor resection	case report	14 (mixed tumors)		x	2000-2012	postoperative hemorrhage	Surgery	2-3 hours	incidence	2 cases of CP, 50 yo male and 56 yo male; no surgery history, no hydrcephalus; right frontotemporal surgical approach, no ventricular drainage, first case no cerebral hernia, second case cerebral hernia
Zhang et al. 2023	Neurosurgical managament of proton beam therapy-induced moyamoya syndrome	retrospective case review	10 (mixed tumors)	x		2009-2022	Moyamoya syndrome syndrome	Radiation	median 20 (14.5-41) months	incidence	6 patients with CP and Moyamoya, 5 cases with TIA, two cases with stroke, medical or surgical treatment, all patients pituitary dysfunction, vulnerable to fluid and eletrolyte disturbances and vulnerable to dehydration and ischemia

Zuccaro et al. 1996	Complications in paediatric craniopharyngioma treatment	retrospective cohort study	48	x		1988-1994	postsurgical subdural haematomas, vascular complications	not specified	na	incidence	1 patient died in the immediate postoperative period due to onjury to the internal carotid artery, one received intracystic yttrium-90 as initial treatment, developed hemiplagia, moyamoya syndrome as bilateral carotid stenosis, after surgery second recurrence, bilateral cerebral infarction, patient died; third patient developed internal carotid artery soasm, cerebral infarction and hemiparesis following totoal tumor removal; subdural haematomas: 8 patients required treatment for intracranial hypertension, five shunting, subdural haematomas were eveacuated in all patients; shunt removed in three

Table S2: Details on studies assessing surgery and vascular complications.

Author	Year	Vascular complication	Approach	Note
Probst et al.	1992	cardiac arrest	transcranial	
Lakhanpal	1995	carotid pseudoaneurysm	n.s.	
Cho et al.	2012	cerebral infarction	transcranial	
Fischer et al.	2004	cerebral infarction in left MCA territory and meningitis	transcranial	
Nash et al.	2016	Cerebral vasospasm	trans-sphenoidal	
Elliott and Wisoff	2010	FDCA	transcranial	
Hoffmann et al.	2016	FDCA	transcranial	78.6% of patients with FDCA had transcranial approach
Aboukais et al.	2021	FDICA	transcranial	
Sutton et al.	1994	Fusiform dilatation of carotid artery (FDCA)	transcranial	
Sutton et al.	1991	Fusiform dilatation of carotid artery (FDCA)	transcranial	
Li et al.	2015	Fusiform dilatation of carotid artery (FDCA)	transcranial	
Linfante et al.	2008	Fusiform dilatation of carotid artery (FDCA)	n.s.	
Liu et al.	1991	Fusiform dilatation of carotid artery (FDCA)	transcranial	
Nagata et al.	2010	Fusiform dilatation of carotid artery	transcranial	

		(FDCA)		
Wang et al.	2016	Fusiform dilation of the internal carotid artery (FDICA)	transcranial	
Tirakotai et al.	2002	Fusiform diltatation of the internal carotid artery (FDICA)	transcranial	
Macdonald	1997	hemorrhage	transcranial	
Yu et al.	2016	postoperative hemorrhage	transcranial	
Cavallo et al.	2013	postsurgical complications: hemorrhage, CSDH	trans-sphenoidal	
Lambert et al	2022	pulmonary embolism (following deep venous thrombosis)	transcranial	
Boekhoff et al.	2021	Stroke	both	76% of patients with CI had fronto-temporal approach
Fukuhura et al.	2017	subdural hemorrhage	trans-sphenoidal	
Jamaluddin et al.	2020	supra-clinoid carotid aneurysm	transcranial	
Singh et al.	2017	Vasospasm	transcranial	
Koumas et al.	2019	Vasospasm, Stroke	trans-sphenoidal	10% of patients had vasospasm/stroke
Ricarte et al.	2015	Vasospasm, Stroke	trans-sphenoidal	
Qiao et al.	2023	venous thromboembolism (PE and DVT)	both	higher risk in patients with transcranial approach (OR = OR 2.78, p = 0.017)

Table S3: Details on studies assessing radiotherapy and vascular complications.

**Author**	**Year**	**Vascular complication**	**Type of Radiotherapy**	**Dose**	**Notes**
Sandvik et al.	2019	Fusiform aneurysm, Moyamoya syndrome	proton therapy, stereotactic gamma knife	54 Gy	
Boon et al.	2016	hemipontine infarct	n.s.	n.s.	
Pereira Cruz	2002	ICA aneurysm and vasculopathy	cranial radiotherapy	54 Gy	
Strenger et al.	2008	intracerebral cavernoma	photon therapy	55 Gy	
Visser et al.	2010	MCA infarct after Moyamoya disease	photon and proton therapy	50 Gy	unclear which therapy was used in patient with MCA infarct
Lee et al.	2017	Moyamoya syndrome	proton therapy	54 Gy	
Reynolds et al.	2016	Moyamoya syndrome	proton therapy	54 Gy	
Almeida et al.	2019	Moyamoya syndrome	n.s.	n.s.	
Bitzer et al.	1995	Moyamoya syndrome	cranial radiotherapy	50 Gy	
Bolsi et al.	2020	Moyamoya syndrome	proton therapy	54 Gy	2 out of 16 presented with vasculopathy after PT
Gleeson et al.	2008	Moyamoya syndrome	external beam	n.s.	
Kato et al.	2019	Moyamoya syndrome	cranial radiotherapy	50 Gy	
Wu et al.	2016	Moyamoya syndrome	cranial radiotherapy	54 Gy	

Zhang et al.	2023	Moyamoya syndrome	proton therapy	n.s.	
Ravindra	2021	Moyamoya, stroke	proton therapy	54 Gy	2 patients with progressive vasculopathy after proton beam therapy
Kralik et al.	2017	radiation-induced large vesselcerebral vasculopathy (RLVCV)	proton therapy	54.5 Gy	5 of 75 (6.7%)
Lo et al.	2016	stroke	n.s.	n.s.	
Mitchell et al.	1991	stroke	suprasellar RT	6174 rad	carotid arteries, cricle of Willis in the radiation field
Indelicato et al.	2017	stroke	proton therapy	54 Gy	
Lee et al.	2011	TIA and Moyamoya syndrome	whole brain RT	54 Gy	
Edmonston et al.	2022	vasculopathy	focal	54-55.8 Gy	8.5% had vasculopathy; larger planning target volume was associated with an increased risk of vasculopathy
Liu et al.	2009	vasculopathy	n.s.	52.2 Gy	
Lucas et al.	2022	vasculopathy	proton therapy	54.1 Gy	36.2% with new post-RT vasculopathies
Merchant	2023	vasculopathy	proton therapy	54 Gy	similar numbers of vasculopathies in photon vs. Proton
Sarkar et al.	2022	vasculopathy	conventional, 3D-conformal, fractionated stereotactic	50.4-54 Gy	two patients with vasculopathies had salvage RT
Hall et al.	2018	vasculopathy and stroke	proton therapy	54 CGE	3-year cumulative rates of any vasculopathy 6.4% and serios vasculapathy 2.6%; 1.2% stroke
